# Clinical and Radiographic Features of Pycnodysostosis with Emphasis on Dentofacial Problems

**DOI:** 10.1155/2017/4352485

**Published:** 2017-11-26

**Authors:** Hossein Aghili, Seyed Mohammad Ali Tabatabaei, Mahdjoube Goldani Moghadam

**Affiliations:** ^1^Faculty of Dentistry, Shahid Sadoughi University of Medical Sciences, Yazd, Iran; ^2^Faculty of Dentistry, Birjand University of Medical Sciences, Birjand, Iran

## Abstract

Pycnodysostosis (PDO) is a rare genetic disorder characterized by cathepsin K deficiency which plays an important role in bone metabolism. Among clinical features of this disease which are mainly caused by altered bone remodeling are craniofacial abnormalities such as hypoplastic maxilla and obtuse gonial angle which consequently lead to respiratory insufficiency in forms of pharyngeal narrowing and severe snoring. In this paper, another case of this rare disorder is presented along with a review on etiology and management issues of respiratory insufficiency in these patients.

## 1. Introduction

Pycnodysostosis (PDO) is a rare genetic disorder with less than 200 patients affected worldwide [1]. It was first described as a distinct entity by Maroteaux and Lamy in 1962 [2]. The responsible gene is *CTSK* which maps to chromosome 1q21 and encodes cathepsin K, a lysosomal cystine protease. Impaired function of this critical enzyme for bone remodeling and resorption by osteoclasts leads to the fragile and sclerosing nature of the bone in affected patients [3]. The mode of inheritance is compatible to autosomal recessive [4].

Common characteristics of the disorder include increased bone density, short stature, cranial dysplasia, clavicular dysplasia, frequent fractures, delayed suture closure, dysplasia of terminal phalanges, frontal and occipital bossing, proptosis, blue sclera, and beaked nose [5, 6]. Hypoplastic maxilla and obtuse gonial angle are the most common jaw abnormalities. Midface retrusion often results in skeletal class III pattern [7]. In intraoral clinical and radiographic evaluation, grooved palate, severe crowding, poor oral hygiene, periodontal problems, delayed exfoliation of primary teeth, and eruption of permanent teeth are usually evident [8, 9]. In addition, dental abnormalities such as enamel hypoplasia, obliteration of pulp chambers, and hypercementosis can be seen [10].

In children with PDO, special dental care is needed with greatest emphasis on preventive treatments. As stated earlier, caries and poor oral hygiene are frequently observed which may necessitate extractions. The extraction of teeth may become a challenge regarding decreased bone remodeling and osteosclerosis, and there is a potential for postextraction osteomyelitis to develop. Reports of osteomyelitis of jaws in PDO patients exist in the literature [8, 11–13]. Furthermore, orthodontic and orthognathic treatments which are dependent on normal osteoclastic function and bone remodeling are controversial issues in PDO [14].

Respiratory insufficiency in forms of snoring and obstructive apnea is also common among PDO patients [7]. Retrognathia and glossoptosis which are the consequences of maxillary hypoplasia and flattened mandibular angle as well as long soft palate which is commonly associated with the disorder are explanations for pharyngeal narrowing and severe snoring in PDO [15].

The objective of this paper is to report on a case of pycnodysostosis with severe snoring and to discuss etiology and management issues of respiratory insufficiency in these patients. Little attention has been paid to respiratory problems of PDO sufferers and the craniofacial related causative factors of the problems are not sufficiently studied.

## 2. Case Report

**Figure 1:**
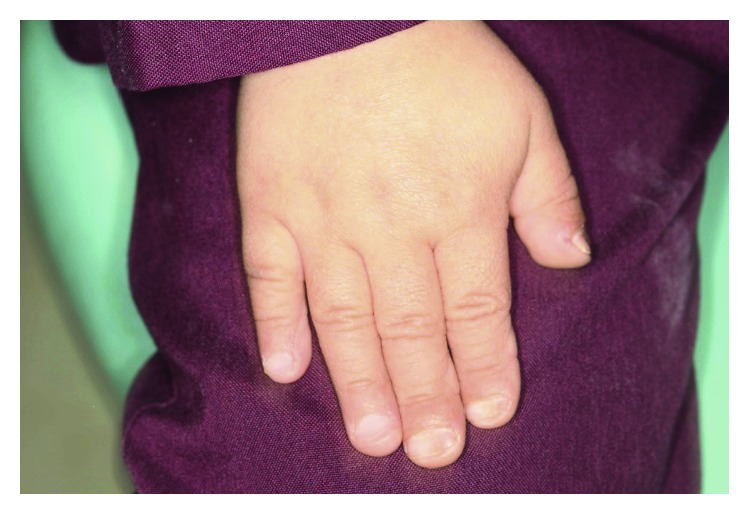
Abnormalities in the fingers and skin in a patient suffering from PDO.

A 5-year 8-month-old girl was referred to the department of orthodontics with complaint of severe snoring. The diagnosis of PDO had been made through a complete medical survey at 4 years of age. No parental consanguinity was present. The patient presented typical characteristics of the disorder including retrognathia, beaked nose, frontal bossing, and short fingers with wrinkled skin (Figure 1).

**Figure 2:**
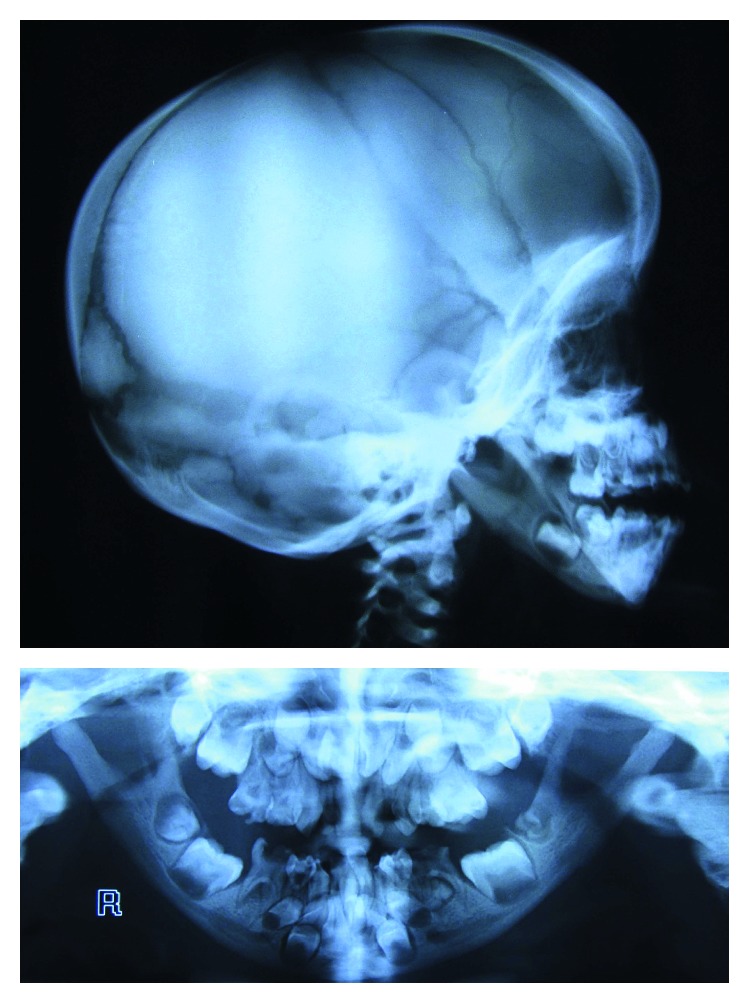
Open fontanels and obtuse mandibular angle in PDO.

**Figure 3:**
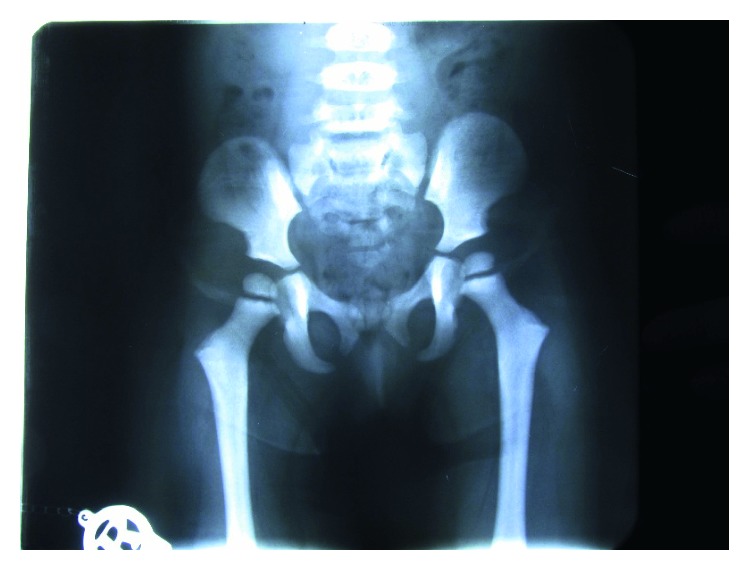
Coxa valga deformity in PDO sufferers.

The patient's radiographs revealed a very obtuse mandibular angle, general increase in bone density, and open fontanels and sutures (Figure 2). The radiologist's report included general increase in bone density, craniofacial disproportion, open fontanels, wormian bones, hypoplasia of clavicle, hypoplastic distal phalanges with absence of distal tufts, mild coxa valga deformity (a deformity of the hip where the angle formed between the head and neck of the femur and its shaft is increased, usually above 135°) (Figure 3), and spondylosis of L4.

She presented severe upper airway obstruction, and according to the parents, she snored profusely during sleep. The patient had undergone one night of monitoring with polysomnography to evaluate the severity of the breathing disorder. The polysomnography consisted of respiratory analysis, evaluation of O_2_ saturation, heart rates, periodic limp movement, snore analysis, microarousal, and systolic blood pressure. The results of the sleep study in summary showed severe snoring (1952 times and snore index of 357.6).

**Figure 4:**
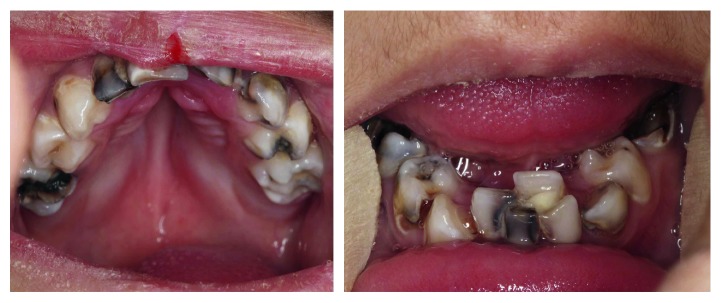
Intraoral features of PDO.

In intraoral examination, a grooved palate and severe crowding were noted. The patient's oral hygiene was poor, and caries and gingivitis were evident (Figure 4).

Cephalometric evaluation revealed retrognathic maxilla and mandible, long soft palate, severely reduced pharyngeal airway space between the uvula and posterior pharyngeal wall (PAS-UP) and also between the tongue and posterior pharyngeal wall (PAS-TP), and decreased posterior airway space (PAS). The variables which were evaluated are as follows:

  SNA: the sella-nasion point A angle
  SNB: the sella-nasion point B angle
  MaxL (maxillary length): the distance between ANS and PNS
  Ar-Gn: the distance between a point at the junction of the posterior border of the ramus and the inferior border of the posterior cranial base (Ar) and Gn
  PAS: measured between the posterior pharyngeal wall and the dorsum of the tongue on a line joining the gonion to the B point
  Ba-PNS: the distance between the lowest point on the anterior rim of foramen magnum (Ba) and PNS
  Soft palate length: the distance from PNS to the tip of the soft palate or uvula (UP)
  C3-Me: the distance between the most superior anterior point on the third cervical vertebra (C3) and the Me
  Hy-ML: the perpendicular distance from the most anterior superior point of the hyoid (Hy) to the mandibular plane (ML)
  PAS-UP: the perpendicular distance between the posterior pharyngeal wall (PAS) and soft palate or uvula tip (UP)
  PAS-TP: the perpendicular distance between the posterior pharyngeal wall (PAS) and back of the tongue (TP).
  Cephalometric findings are shown in Table 1.

## 3. Discussion

Muto and colleagues have suggested that respiratory insufficiencies in forms of snoring or obstructive sleep apnea are common and underappreciated features of PDO [7]. In 1974, Nielsen reported six cases of PDO who had presented with long soft palate in addition to typical features of the disorder [16]. The etiology of respiratory problems had not been sufficiently studied, and therefore the mechanisms of upper airway obstruction had remained unclear until recently. Later, it was proposed that retrognathia which is a common finding in PDO sufferers leads to glossoptosis and consequently narrowing of the airway space [7]. Furthermore, Muto and colleagues found that the measurements of Ba-PNS, C3-Me, and Hy-ML which form a frame encompassing the pharyngeal airway space were small in their PDO cases [7]. We also obtained similar results from the cephalometric study in our case. An interesting finding which has been reported in several cases of PDO patients is long soft palate in spite of maxillary and mandibular deficiencies [7, 16–18]. The case presented in this paper was another example with similar findings: a long soft palate along with hypoplasia of maxilla and mandible. In overall, airway obstruction in PDO sufferers has been attributed to the long soft palate, small skeletal frame around the pharyngeal airway space, maxillary and mandibular deficiencies, long distance between the hyoid and mandible, and posteriorly situated maxilla [7]. The data obtained from our case concur with these findings.

In the review of treatment options for management of different aspects of the disorder including esthetic and functional demands of the patients, no straightforward protocol can be recommended. The efficacy and safety of orthodontic and orthopedic treatments are issues of debate. Orthodontic and orthopedic movements are completely dependent on bone remodeling and normal function of osteoclasts. As stated earlier, the impaired function of cathepsin K results in dysfunction of osteoclasts and consequently reduction in bone resorption and remodeling and increased osteosclerosis and compromises orthodontic and orthopedic treatments [4].

Surgical approaches are subjected to the increased risk of developing osteomyelitis as a result of decreased blood supply of the sclerotic bone [19, 20]. However, few cases of skeletal facial surgery have been reported [15, 20–22]. Teissier and coworkers presented a case of PDO who was a 3-year 6-month-old boy suffering from severe snoring as a result of hypoplastic mandible. They treated the patient with a bilateral rib graft which caused enlargement of the pharynx and significant reduction in the snoring. The authors suggested that rib graft was an ideal treatment modality for such patients [15]. Hernández-Alfaro and colleagues have described an 18-year-old girl with diagnosis of PDO and chief complaint of facial appearance who has been treated with bimaxillary orthognathic surgery. The authors reported good and stable results in terms of both esthetic and occlusion [20]. Another report was pertained to a case of PDO who was a 15-year-old girl in whom maxillary advancement and inferior positioning by distraction osteogenesis were performed. The authors observed a reasonably stable maxillary advancement and suggested that extraoral distraction osteogenesis should be considered in the treatment of PDO patients with maxillary hypoplasia [21].

The PDO sufferers usually presented poor oral hygiene and extensive caries [8, 9]. Tooth extractions in these patients need special care including atraumatic technique and proper asepsis to prevent developing of postextraction osteomyelitis [8, 11, 13]. Therefore, regarding difficulties in management of caries, the importance of preventive care in PDO patients should be well understood by pediatric dentists.

The dental managements in these patients are mainly focused on restoring the decayed teeth and put the patient on a strict prevention program. Selective extractions to relieve crowding and using oral appliances to manage the pertained deformities are treatment procedures which are performed in each case regarding his/her individual problems.

In management of respiratory insufficiency in PDO, treatments should address the etiology of the problem. As stated earlier, presence of long soft palate and maxillary and mandibular deficiencies are the main causes of upper airway obstruction in the sufferers [7]. To have a better knowledge of appropriate treatment modalities, it is necessary to locate the site of obstruction and accordingly plan a treatment strategy. Obstruction may occur at three levels, which each of them require specific treatments including nasal obstruction, oropharyngeal obstruction, and hypopharyngeal obstruction. Nasal polyps, deviated nasal septum, and hypertrophic inferior turbinates can cause nasal obstruction. More common sites are obstructions at the oropharyngeal and hypopharyngeal levels. Oropharyngeal obstruction results from problems at the soft palate, pharynx, and tonsillar pillars. Increased tongue mass volume or its retro position produces hypopharyngeal obstruction [23].

In order to improve the problem of long soft palate, which is among the most common causes of snoring, several methods have been used including uvulopalatopharyngoplasty (UPPP) [24], laser-assisted uvulopalatoplasty (LAUP) [25], radiofrequency (RF) tissue ablation [26], and the placement of polyethylene terephthalate (PET) palatal implants [27]. Since the explanation of technical details of these methods is beyond the goals of this paper, the reader is referred to the references given for each of methods.

Other sources of respiratory problems are maxillary and mandibular deficiencies. As discussed earlier, treatment of severe snoring and pharyngeal narrowing due to hypoplastic mandible by using bilateral rib graft has been reported [15]. Microimplant-based mandibular advancement therapy for treatment of snoring and obstructive sleep apnea has been performed. Extraoral anchorage was provided with a customized face mask to which microimplants were connected. Mandibular advancement by this method resulted in reduction in obstructive sleep apnea and snoring symptoms [28]. Oral appliance therapy has been said to advance mandible and tongue and consequently increases the size of pharyngeal lumen, but its efficacy is variable and dependent on appliance design and host factors. As high as thirty-four percent contraindications due to dental factors, insufficient teeth and periodontal disease have been reported, and it has been said to impose several side effects ranging from occlusal alteration to TMJ and muscular pain [29, 30]. Distraction osteogenesis can provide a slow and controlled advancement in the jaws and has been reported to be a reliable surgical method in improving the narrow upper airway especially in patients with severe maxillomandibular deformities [31]. Surgical advancement of the jaws has also been reported as the most definitive, successful, and predictable surgical procedure in the management of obstructive sleep apnea other than tracheotomy [23]. However, it is an aggressive surgery, and early postoperative edema and well-known tendency for relapse are serious concerns [23].

## 4. Conclusion

In patients with craniofacial anomalies affecting airway anatomy such as PDO, treatment of respiratory problems should be considered as a priority. Untreated disordered breathing especially in children causes sleepiness after awaking, tiredness, and attention problems and adversely affects their learning abilities. Regarding problems associated with PDO which make many of the aforementioned treatment modalities difficult for them, the authors suggest special attention to preventive care in the patients specially in pediatric PDO sufferers and to initially try less invasive therapies for treatment of disordered breathing, such as face mask therapy which itself is a type of distraction osteogenesis and also can be modified to be used as an extraoral anchorage for mandibular advancement; however, the efficacy of these methods needs to be studied in the affected patients.

## Figures and Tables

**Table 1:** Cephalometric findings.

Variables	Normal value	Case
SNA	82°	74°
SNB	80°	69°
MaxL	51 mm	39 mm
Ar-Gn	116 mm	61 mm
PAS	11 mm	1 mm
Ba-PNS	46 mm	31 mm
Soft palate length	36 mm	40 mm
C3-Me	85 mm	47 mm
Hy-ML	11 mm	15 mm
PAS-UP	11 mm	1.5 mm
PAS-TP	13 mm	3.5 mm

## References

[1] Ornetti P., Prati C., Fery-Blanco C., Streit G., Toussirot E., Wendling D. (2008). Pedicle stress fracture: an unusual complication of pycnodysostosis. *Clinical Rheumatology*.

[2] Maroteaux P., Lamy M. (1962). Deux observations d’une affection osseuse condensante la pycnodysostose. *Archives Francaises de Pediatrie*.

[3] Gelb B. D., Edelson J. G., Desnick R. J. (1995). Linkage of pycnodysostosis to chromosome 1q21 by homozygosity mapping. *Nature Genetics*.

[4] Soliman A. T., Ramadan M. A., Sherif A., Aziz Bedair E. S., Rizk M. M. (2001). Pycnodysostosis: clinical, radiologic, and endocrine evaluation and linear growth after growth hormone therapy. *Metabolism*.

[5] Maroteaux P., Lamy M. (1962). La pycnodysostose. *Presse Medicale*.

[6] Andren L., Dymling J. F., Hogeman K. E., Wendeberg B. (1962). Osteopetrosis acro-osteolytica: a syndrome of osteopetrosis, acro-osteolysis and open sutures of the skull. *Acta Chirurgica Scandinavica*.

[7] Muto T., Yamazaki A., Takeda S., Tsuji Y., Shibata T. (2005). Pharyngeal narrowing as a common feature in pycnodysostosis—a cephalometric study. *International Journal of Oral and Maxillofacial Surgery*.

[8] Bathi R. J., Masur V. N. (2000). Pyknodysostosis–a report of two cases with a brief review of the literature. *International Journal of Oral and Maxillofacial Surgery*.

[9] Hunt N. P., Cunningham S. J., Adnan N., Harris M. (1998). The dental craniofacial, and biochemical features of pyknodysostosis a report of three new cases. *Journal of Oral and Maxillofacial Surgery*.

[10] Landa S., Esteban S., Montes E., Santamaria J., Vitoria A., Santolaya J. M. (2000). Maxillofacial alterations in a family with pycnodysostosis. *Medicina Oral*.

[11] van Merkesteyn J. P., Bras J., Vermeeren J. I., van der Sar A., Statius van Eps L. W. (1987). Osteomyelitis of the jaws in pycnodysostosis. *International Journal of Oral and Maxillofacial Surgery*.

[12] Kato H., Matsuoka K., Kato N., Ohkubo T. (2005). Mandibular osteomyelitis and fracture successfully treated with vascularised iliac bone graft in a patient with pycnodysostosis. *British Journal of Plastic Surgery*.

[13] Iwu C. O. (1991). Bilateral osteomyelitis of the mandible in pycnodysostosis. A case report. *International Journal of Oral and Maxillofacial Surgery*.

[14] O’Connell A. C., Brennan M. T., Francomano C. A. (1998). Pycnodysostosis: orofacial manifestations in two pediatric patients. *Pediatric Dental Journal*.

[15] Teissier N., Jacquemont M. L., Blancal J. P., Elmaleh-Bergès M., van Den Abbeele T., Bennaceur S. (2009). Severe snoring in a child with pycnodysostosis treated with a bilateral rib graft. *Cleft Palate-Craniofacial Journal*.

[16] Nielsen E. L. (1974). Pycnodysostosis—six cases with new symptoms and an autopsy. *Acta Paediatrica Scandinavica*.

[17] Fonteles C. S., Chaves C. M., Da Silveira A., Soares E. C., Couto J. L., de Azevedo Mde F. (2007). Cephalometric characteristics and dentofacial abnormalities of pycnodysostosis: report of four cases from Brazil. *Oral Surgery, Oral Medicine, Oral Pathology, Oral Radiology, and Endodontics*.

[18] Alibhai Z. A., Matee M. I., Chindia M. L., Moshy J. (1999). Presentation and management of chronic osteomyelitis in an African patient with pycnodysostosis. *Oral Diseases*.

[19] Schmitz J. P., Gassmann C. J., Bauer A. M., Smith B. R. (1996). Mandibular reconstruction in a patient with pyknodysostosis. *Journal of Oral and Maxillofacial Surgery*.

[20] Hernández-Alfaro F., Arenaz Búa J., Serra Serrat M., Mareque Bueno J. (2011). Orthognathic surgery in pycnodysostosis: a case report. *International Journal of Oral and Maxillofacial Surgery*.

[21] Nørholt S. E., Bjerregaard J., Mosekilde L. (2004). Maxillary distraction osteogenesis in a patient with pycnodysostosis: a case report. *Journal of Oral and Maxillofacial Surgery*.

[22] Polley J. W., Figueroa A. A. (1999). Maxillary distraction osteogenesis with rigid external distraction. *Atlas of the Oral and Maxillofacial Surgery Clinics of North America*.

[23] Ephros H. D., Madani M., Yalamanchili S. C. (2010). Surgical treatment of snoring & obstructive sleep apnoea. *Indian Journal of Medical Research*.

[24] Fujita S., Conway W., Zorick F., Roth T. (1981). Surgical correction of anatomic abnormalities in obstructive sleep apnea syndrome: Uvulopalatopharyngoplasty. *Otolaryngology-Head and Neck Surgery*.

[25] Kamami Y. V. (1990). Laser CO_2_ for snoring: preliminary results. *Acta Oto-Rhino-Laryngologica Belgica*.

[26] Ellis P., Williams J. E., Shneersan J. (1993). Surgical relief of snoring due to palatal flutter: a preliminary report. *Annals of the Royal College of Surgeons of England*.

[27] Madani M. (2007). Palatal implants for treatment of habitual snoring; techniques, indications and limitations. *Atlas of the Oral and Maxillofacial Surgery Clinics of North America*.

[28] Ngiam J., Kyung H. M. (2012). Microimplant-based mandibular advancement therapy for the treatment of snoring and obstructive sleep apnea: aprospective study. *Angle Orthodontist*.

[29] Ahrens A., McGrath C., Hagg U. (2010). Subjective efficacy of oral appliance design features in the management of obstructive sleep apnea: a systematic review. *American Journal of Orthodontics and Dentofacial Orthopedics*.

[30] Petit F. X., Pepin J. L., Bettega G., Sadek H., Raphael B., Levy P. (2002). Mandibular advancement devices: rate of contraindications in 100 consecutive obstructive sleep apnea patients. *American Journal of Respiratory and Critical Care Medicine*.

[31] Xiaofeng L., Yousheng T., Guofang S., Ming Z., Qingyun L., Weiliu Q. (2008). Distraction osteogenesis for the patients of OSAS with craniomaxillomandibular deformities. *Journal of Oral and Maxillofacial Surgery*.

